# Assessment of Knowledge, Attitudes, and Practices towards New Coronavirus (SARS-CoV-2) of Health Care Professionals in Greece before the Outbreak Period

**DOI:** 10.3390/ijerph17144925

**Published:** 2020-07-08

**Authors:** Dimitrios Papagiannis, Foteini Malli, Dimitrios G. Raptis, Ioanna V. Papathanasiou, Evangelos C. Fradelos, Zoe Daniil, Georgios Rachiotis, Konstantinos I. Gourgoulianis

**Affiliations:** 1 Faculty of Nursing, University of Thessaly, 41500 Larissa, Greece; dpapajon@gmail.com (D.P.); iopapathanasiou@yahoo.gr (I.V.P.); evagelosfradelos@hotmail.com (E.C.F.); 2 Respiratory Medicine Department, Faculty of Medicine, University of Thessaly, 41100 Larissa, Greece; raptisdmed@gmail.com (D.G.R.); zdaniil@med.uth.gr (Z.D.); kgourg@med.uth.gr (K.I.G.); 3 Department of Hygiene and Epidemiology, Medical Faculty, School of Health Science, University of Thessaly, 41100 Larissa, Greece; grachiotis@gmail.com

**Keywords:** SARS-CoV-2, health care professionals, knowledge, attitudes and practices, Greece, COVID-19

## Abstract

Introduction: The ongoing severe acute respiratory syndrome (SARS)-CoV-2 pandemic has expanded globally. The aim of the current study is to investigate the knowledge, attitudes, and practices (KAP) of health care professionals in Greece towards SARS-CoV-2. Methods: From 10–25 February 2020, 500 health care workers were approached. Knowledge, attitudes, and practices towards SARS-CoV-2 were assessed via a personal interview questionnaire. For knowledge, each correct answer was given 1 point; attitudes, or concerns aimed at prevention of SARS-CoV-2 infection, and practices, or behaviors towards performing preventive practices, were assigned 1 point each. Points were summed and a score for each category was calculated. Results: A total of 461 health care workers returned the questionnaire and were included in the analysis (mean age ± SD: 44.2 ± 10.78 years, 74% females). The majority were nurses (47.5%), followed by physicians (30.5%) and paramedics (19%). The majority of subjects (88.28%) had a good level of knowledge (knowledge score equal to 4, or more). The majority of participants (71%) agreed with the temporary traveling restrictions ban. The uptake of a future vaccine against SARS-CoV-2 was estimated at 43%. Knowledge score was significantly associated with both attitudes score (*p* = 0.011) and practices score (*p* < 0.001), indicating that subjects with a high knowledge score demonstrated a more positive perception on preventive measures and would practice more preventive measures. Attitudes score was significantly associated with practices score (*p* = 0.009) indicating that subjects with a higher attitudes score are more likely to perform practices towards the prevention of SARS-CoV-2 transmission. Conclusion: There is a high level of knowledge concerning SARS-CoV-2 pandemic among Greek health care workers and this is significantly associated with positive attitudes and practices towards preventive health measures. The high level of knowledge of health professionals about SARS-CoV-2 may have contributed considerably to the successful management of the pandemic in Greece. Tailored educational campaigns aiming to increase the proportion of health care workers willing to accept a potential SARS-CoV-2 vaccine could be of paramount importance in future proactive vaccine educational campaigns.

## 1. Introduction

The novel coronavirus disease 2019 (COVID-19) has spread into four continents, and human-to-human transmission has been confirmed [1,2]. Roughly 49 countries had confirmed cases of Severe Acute Respiratory Syndrome (SARS)-CoV-2 infection by the 29th of February 2020, and the overall numbers are growing [3]. At the end of February, most of the cases were in tourists from China or people who had recently been to China. Since then, countries have reported cases in which a person who had not traveled to China contacted the virus from someone who had. Some of those cases are spreading the virus as well, and the incubation period has been reported to be 5.2 days, although studies have suggested that it may be as long as 14 days [4]. After SARS and Middle East respiratory syndrome (MERS) outbreaks, and the 2009 influenza H1N1 pandemic, the risk of a spread of a new pandemic has reemerged by a new strain of coronavirus that has not been previously identified in humans. The World Health Organization (WHO) declared the SARS-CoV-2 outbreak as a Public Health Emergency of International Concern on the 30th of January 2020 and then a pandemic on the 11th of March 2020, as a result of the worldwide spread of COVID-19 [5]. According to the world meter (updated: July 2, 2020), SARS-CoV-2 has affected 213 countries and territories around the world. To date, 10,884,519 cases have been reported worldwide followed by 520,604 deaths and 6,085,813 recovered cases. In Greece, there have been 3589 confirmed cases with 193 deaths [6].

The World Health Organization (WHO) rapidly developed advice to meet the need for recommendations of safe home care for patients with suspected SARS-CoV-2 infection presenting with mild symptoms as well as public health measures related to management of asymptomatic contacts [7], while the Centers for Disease Control and Prevention (CDC) also published a guide with criteria for the evaluation of patients under investigation for SARS-CoV-2 [8]. Health care workers in particular are extremely vulnerable to SARS-CoV-2 infection since they are frequently in contact with COVID-19 patients [9]. In some countries as much as 10% of health care workers are infected with SARS-CoV-2 and the WHO has outlined the need for training of health care workers in order to reduce the rates of infection [8] The Greek CDC also developed special recommendations for health care workers (HCWs) [10]. Despite the fact that health care workers play a central role in the response to COVID-19, to our knowledge there is very limited information on the knowledge, attitudes, and practices of health care workers towards SARS-CoV-2.

The aim of this study is to investigate the knowledge, attitudes, and practices of health care workers towards the COVID-19.

## 2. Methods and Instruments

From 10–25 February 2020, a structured, self-administered, anonymous questionnaire was distributed via personal interviews to a convenient sample of 500 health care workers in five public hospitals during two consecutive days at work. The anonymous questionnaire was composed after taking into consideration the knowledge and practices from the health care professionals in central Greece after the international guidelines were published by WHO and local directions by the Hellenic public health authorities (Greek CDC and Ministry of Health). Health care professionals from five public hospitals from Thessaly (one tertiary and four secondary) participated in the present study. Ethical approval from the scientific committee of the University Hospital of Larissa (protocol number 5985-7/2/2020) was obtained. All participants provided verbal and written consent.

Three different groups of health care workers (i.e., physicians, nurses, and paramedical staff) were selected as survey subjects. The anonymous questionnaire was distributed to a convenience sample of 500 health care workers in five public hospitals and was completed voluntarily by 461 health care workers (92.2% response rate). The questionnaire included 18 questions for the assessment of knowledge, attitudes, and practices and demographics (including age, gender, district of residence, occupation, and previous work experience duration) (see online Supplementary Material). Questions assessing recent travel history and the personal perception of the level of knowledge concerning COVID-19 were included in the questionnaire. Knowledge was assessed with eight questions. Information for knowledge about SARS-CoV-2 was evaluated from the reports and the directions published by the international and local health authorities. Attitudes and risk perception towards COVID-19 (concerning preventive measures, preference for information source) and practices (concerning preventive measures, personal hygiene practices) were assessed. A five-point Likert-type scale was used to ascertain the level of agreement or disagreement for the questions (from 1 to 5; 1 = fully disagree, 2 = disagree, 3 = uncertain, 4 = agree, 5 = fully agree). For some questions, response options included “yes”, “no” or “uncertain” (see online Supplementary Material). Scores for the different measures assessed (i.e., knowledge, attitude, and practice) were calculated as follows: for knowledge, each correct answer was given 1 point and an incorrect answer was given 0 points. For attitude, concerns aimed at prevention of SARS-CoV-2 infection were assigned 1 point. For practices, behaviors towards performing preventive practices were given 1 point. Points were summed and a score for each category (knowledge, attitude, and practice) was calculated. Especially for the knowledge score, respondents with a score of 1 to 4 were further categorized as “poor level of knowledge” and those with a score of 4 to 8 as “good level of knowledge”.

The questionnaire was designed and adjusted by the authors for knowledge, attitude, and practice (KAP) study. A pilot test of the first draft of the questionnaire took place in two primary health centers and assessed the ability to complete the questionnaire. The time spent on the completion of the questionnaire was approximately 10–20 min. The results of the pilot testing were used in order to further modify the questionnaire and were excluded from the final analysis.

## 3. Statistical Analysis

Absolute (*n*) and relative frequencies (%) were presented for qualitative variables and mean (±SD) were used for continuous variables. Normal distribution was assessed by the Kolmogorov–Smirnov test. Comparison between groups was performed with the use of Student’s *t*-test or Mann–Whitney U test according to variable distribution. Chi-squared test was used for categorical variables. One-way analysis of variance (ANOVA) or Kruskal-Wallis were used to compare more than two groups according to variable distribution. Statistical analysis was performed using the SPSS 16 statistical package (SPSS, Chicago, IL, USA). Statistical significance was defined as *p*-value < 0.05.

## 4. Results

Mean age of the study subjects was 44.2 ± 10.77 years (Table 1). Of the 461 participants, 119 were male (26%) and 341 were female (74%). The distribution according to work status of the participants was as follows: 47.5% were nurses, 30.5% were physicians, and 19% were paramedic staff. Fifteen participants did not report their work position. All of the participants were of Greek origin. Mean work experience of the study participants was 17.9 ± 11.89 years. Of the respondents, 17.2% had traveled abroad in the previous six months and 23.4% reported recent travel of a close family member. The vast majority of participants (99%) reported that they are aware of the COVID-19 outbreak. The majority (69.8%) of respondents received information from TV/radio, 63% from Internet/web pages/blogs, 28.1% from a physician, and 20.6% from the web page of the Greek CDC. Of the respondents, 76% reported that they knew WHO recommendations for SARS-CoV-2 and 55% claimed that they had sufficient knowledge for the recommendations published by the Greek health authorities.

### 4.1. Knowledge about SARS-CoV-2 Infection

A total of 50% of participants reported that SARS-CoV-2 can be transmitted through infected foods, 19.9% reported that SARS-CoV-2 is sexually transmitted, and 99% identified the inhalation of respiratory droplets as a mode of transmission (Table 2). The majority of participants (85.4%) agreed that COVID-19 is a cause for serious illness and death, while 88.8% reported that the symptoms of COVID-19 may resemble that of seasonal influenza. In addition, 73% answered that a specific drug therapy for COVID-19 does not exist and 83.5% reported that a vaccine specifically aimed for SARS-CoV-2 is not currently available. Of the subjects included, 23.2% had a knowledge score of 6, 22.8% of 5, 18.5% of 4, 14.5% of 7, 7.7% of 8, 7.3% of 3, 4.1% of 2, 0.9% of 1, and 0.9% of the respondents had a knowledge score of 0. The majority of subjects (88.3%) had a good level of knowledge (knowledge score ≥ 4).

### 4.2. Attitudes about SARS-CoV-2 Infection

Overall, most of the respondents (94%) thought that active personal hygiene measures can reduce the risk of SARS-CoV-2 transmission, and the vast majority (98%) would follow special advice from the hospital infectious committee. Only 43% of the participants mentioned that they would be vaccinated against SARS-CoV-2. The majority (84.8%) displayed a high attitudes score (equal to 2), suggesting a positive attitude concerning the prevention of SARS-CoV-2 infection. Of the remaining, 13.7% presented an attitudes score of 1 and 1.5% a score equal to 0.

### 4.3. Practices about SARS-CoV-2 Infection

Most respondents reported that they were washing their hands often or very often and only 24.9% reported that they washed their hands before and after contact with the patient/patient’s environment (Table 2). Remarkably the majority of the participants (71%) agreed with the ban of traveling to countries with a high number of COVID-19 cases. The majority of respondents (73.8%) displayed a practices score equal with 1 and 24.5% had a score of 2. Only 1.7% of participants had a score of 0, suggesting that only few of the health care workers did not perform preventive practices aimed at SARS-CoV-2.

### 4.4. Subgroup Analysis

Subjects that were aware of WHO guidelines were older than subjects that reported not knowing the guidelines or subjects answering “uncertain” (45.70 ± 10.38 vs. 37.98 ± 10.65 vs. 40.85 ± 10.85 years, respectively, *p* < 0.001) and, in the same context, had more years of work experience (19.05 ± 11.23 vs. 13.51 ± 10.02 vs. 14.25 ± 10.54 years, respectively, *p* < 0.001). Paramedic staff were less often aware of WHO guidelines for COVID-19 (63.95%) compared to nurses (79.06%) and physicians (78.01%) (*p* = 0.019 between groups). Subjects that judged the recommendations by the Greek health authorities as sufficient were older than subjects answering “not sufficient” (5.62 ± 10.55 vs. 42.12 ± 10.81 years, respectively, *p* = 0.005) while those who judged their level of knowledge as sufficient were older than those that judged their level of knowledge as insufficient (45.63 ± 10.72 vs. 42.22 ± 10.57, respectively, *p* = 0.004) and had worked for more years (18.81 ± 11.49 vs. 16.38 ± 10.65 years, respectively, *p* = 0.047).

Table 3 shows the variation of knowledge according to various parameters. Specialties differed significantly in almost all questions concerning transmission. In more detail, 58.2% of nurses compared to 67.9% of physicians and 46.6% of paramedics believed that SARS-CoV-2 can be transmitted sexually (*p* = 0.013) while 31.5% of nurses, 39.6% of physicians, and 23.3% of paramedics thought that SARS-CoV-2 can be transmitted through food consumption (*p* = 0.034). In the same context, 91.2% of nurses identified COVID-19 as a cause of serious illness and death vs. 79.2% of physicians and 83.7% of paramedic staff (*p* = 0.012). As far as symptoms of COVID-19 are concerned, 90.3% of nurses, 93.7% of physicians, and 80.3% of paramedics believed that COVID-19 has similar symptoms to the seasonal flu (*p* = 0.014). When asked if there was an available specific drug therapy for COVID-19, only 68% of nurses identified correctly that there is no disease specific drug compared to 90.8% of physicians and 64% of paramedics (*p* < 0.001). There was a marginal difference between specialties concerning whether hand washing reduces the risk of transmission with 95% of nurses, 97.1% of physicians, and 88.4% of paramedic staff answering correctly (*p* = 0.045). Knowledge level was significantly different between specialties (*p* = 0.009). Finally, we observed a gender difference in the question addressing whether there is a specific drug therapy for COVID-19 where 16.10% of males and 30.8% of females answered “Yes” (*p* = 0.010) and in the question assessing whether an available vaccine for SARS-CoV-2 exists in which 7.5% of males vs. 19.6% of females answered correctly (*p* = 0.008).

Table 4 depicts the association of attitudes towards the prevention of SARS-CoV-2 transmission with several variables. There was a significant difference in gender concerning willingness to be vaccinated against SARS-CoV-2 with more male health care workers reporting that they would be vaccinated for COVID-19 than females (58.5% vs. 39%, respectively, *p* = 0.001). Subjects with fewer work experience were more likely to be vaccinated than participants unwilling to be vaccinated (16.27 ± 11.82 vs. 18.87 ± 10.48 years, respectively, *p* = 0.019). In the same context, significantly fewer nurses (34%) and paramedic staff (43.5%) would agree to vaccinate against SARS-CoV-2 than physicians (60.7%) (*p* < 0. 001). More women than men supported travel bans as a measure to prevent SARS-CoV-2 (74% vs. 62.2%, respectively, *p* = 0.011). There was a significant difference between specialties concerning support of temporary travel ban restrictions with 76.14% of nurses and 76.7% of paramedic stuff in favor of it, compared to only 58.15% of physicians (*p* = 0.001). Additionally, there was a significant difference of attitudes in favor of temporary travel ban restrictions among subjects working in a hospital vs. those working in a primary health care center (72.7% vs. 59.6%, respectively, *p* = 0.036).

Table 5 shows the association of various parameters with practices aimed at preventive measures. Variables that had a significant relationship with practice scores were gender (*p* < 0.001), age (*p* = 0.030), and specialty (*p* = 0.005). Subjects who were willing to follow instructions for COVID-19 prevention had more years of work experience (17.99 ± 11.21 years) compared to subjects that would not follow public instructions (11.0 ± 4.12 years) (*p* = 0.016) (Table 5). Interestingly, only 16% of male health care workers washed their hands after each patient compared to 28% of females (*p* = 0.027). As far as specialties are concerned, 25.80% of nurses compared to 34.2% of physicians and 8.8% of paramedics washed their hands after the care of each patient (*p* < 0.001).

Knowledge score was significantly associated with both attitudes score (*p* = 0.011) and practices score (*p* < 0.001) (Table 6) suggesting that subjects with high knowledge score exhibited a more positive perception on preventive measures and would practice more preventive measures. Attitudes score was significantly associated with practices score (*p* = 0.009), indicating that subjects with higher attitudes score are more likely to demonstrate practices towards the prevention of SARS-CoV-2 transmission (Table 6).

## 5. Discussion

The vast majority of the subjects included in the study had a high level of knowledge concerning SARS-CoV-2 infection and transmission suggesting that most participants had been informed of COVID-19. Despite the high level of knowledge, almost 1 in 4 respondents did not wash their hands before and after touching a patient, and after touching patient surroundings, suggesting that health educational campaigns need to aggressively engage health care practitioners in preventive strategies. More than 80% of subjects identified COVID-19 as a potentially deadly and serious health issue but less than half of them were willing to be vaccinated against SARS-CoV-2, indicating that even if a vaccine is developed early, many health care workers will not choose to be immunized against SARS-CoV-2.

KAP surveys are commonly used to identify knowledge gaps and behavioral patterns in order to implement effective interventions. There is a need for deep understanding and identification of factors that may influence attitudes and practices towards COVID-19. We observed that knowledge scores were high among the participants of the study with only 11.06% of the health care workers exhibiting low knowledge scores, even though our survey was conducted before the WHO recognized the COVID-19 outbreak as a pandemic [5] and just before the first case of COVID-19 was registered in Greece on the 25th of February [11]. Our population consisted of health care workers which may at least explain the high level of knowledge. However, previously published data from a KAP survey during COVID-19 in the general population in China revealed a high rate of correct answers in the knowledge questionnaire that the authors attributed to the high educational level of the participants and the severity of the public health program [12]. Furthermore, previous studies revealed heterogeneous results on the knowledge, attitudes, and practices of health care workers towards Ebola and Zika viruses. In particular, Oladimeji et al. reported satisfactory knowledge of Ebola virus disease but without a corresponding level of good practices among Nigerian health care workers [13].

The knowledge score was significantly associated with attitudes and practices scores. Patients with a high level of knowledge exhibited more positive attitudes and perceptions towards preventive measures and were engaged in more prevention practices. Others have previously reported similar associations when performing KAP surveys in other infectious diseases [14]. Better knowledge may result in positive perceptions and attitudes and therefore in good practices, thus aiding in the prevention and management of infectious diseases. Our study was conducted early during the pandemic and may help to set international public health campaigns priorities in order to address the most misunderstood and hazardous practices.

One of the most disturbing findings of our study was that only 1 in 4 health care practitioners washed their hands after touching a patient, and after touching patient surroundings, despite the fact that 94.1% of the respondents knew that SARS-CoV-2 transmission could be reduced with hand washing. Although the modes of SARS-CoV-2 transmission have not been fully determined, studies have proven that the disease is primarily transmitted when in close contact of a carrier or a patient via respiratory droplets produced with coughing or sneezing [15]. Hand washing is recommended for the general population in order to prevent disease transmission [16]. Soap and water seem to annihilate SARS-CoV-2 like other viruses. For health care practitioners, hand hygiene is mandatory in order to prevent infections, both for oneself and for the patients [17]. Given the low rank of practices towards hand hygiene in our population, the immediate organization of a campaign aimed at health care workers that addresses hand hygiene seems mandatory.

Another important aspect of our study is that very few health care workers (43.3%) would be vaccinated for SARS-CoV-2 if there was an available vaccine. To date, the only available measures for the prevention of transmission in the community is hand washing, respiratory hygiene, social distancing, and self-isolation. Currently, more than 13 candidate vaccines in clinical trials and 129 in preclinical trials are been tested [18]. Given that health care workers cannot perform self-isolation, they are at high risk of getting exposed to SARS-CoV-2 and possibly transmitting the virus to their patients. In Italy, 20% of the health practitioners have been infected, while in China. 3300 health workers have been infected and 22 have died [19]. Access to personal protective equipment for health care workers is a major concern in many countries due to shortages associated with the acceleration of the pandemic. The safety of health practitioners is of great importance and the implementation of a vaccine would aid significantly in this direction. Our results highlight the need of a national strategy and health education program aimed to enhance the immunization of health care workers in order to protect themselves as well as citizens from infection.

More than 3 in 4 respondents were in favor of a travel ban in countries with high number of cases of COVID-19. Our study was performed prior to the implementation of such measures in Greece. Currently many nations have imposed restrictions in traveling as an attempt to slow down the spread of SARS-CoV-2. However, a travel ban in Wuhan delayed the progression of the epidemic in the local community by only few days but by almost 80% on an international scale [20]. Mathematical models have suggested that implementation of a travel quarantine could reduce only modestly the epidemic progression, although greater effects would be expected if there is at least a 50% reduction of transmission in the community.

Our study has several limitations. We acknowledge that the regional sample of the participants is a limitation of the study design. Additionally, the convenience sampling is another shortcoming of our survey. Convenient sampling has disadvantages related to population bias that may limit the extrapolation of the results in the target population. The study employed a convenient sample since time is of essence in COVID-19 research and thus the sample may not be representative of all health care workers. Nevertheless, we believe that the data presented here could be considered as a satisfactory reflection of the knowledge, attitudes, and practices of Greek health care workers regarding SARS-CoV-2 infection prevention, given that our sample included staff from both general and university hospitals. Moreover, Thessaly is a large region in Greece, with almost 10% of the country’s population. KAP studies present a questionnaire-based study and thus, there is a potential for information bias to occur. Another potential limitation of our survey could be that participants’ gender was overwhelmingly female. However, a previous study from the same region reported a similar gender distribution of the health care workforce [21]. Last, we acknowledge the lack of questions addressing the use of personal protective equipment.

## 6. Conclusions

Our study highlights a high level of knowledge concerning SARS-CoV-2 among Greek health care workers and this was significantly associated with positive attitudes and practices towards preventive health measures. The high level of knowledge of health professionals about SARS-CoV-2 may be considered to have contributed considerably to the successful management of the pandemic in Greece. Tailored educational campaigns aimed to increase the proportion of health care workers willing to accept a potential COVID-19 vaccine could be of paramount importance in future proactive SARS-CoV-2 vaccine educational campaigns [22].

## Figures and Tables

**Table 1:** Demographic characteristics of the health care workers.

Characteristics	N/Total (%) or Mean ± SD
Sex	
Male	119/461 (26%)
Female	341/461 (74%)
Age (years)	44.2 ± 10.78
Occupation	
Physician	141/461 (30.5%)
Nurse	219/461 (47.5%)
Paramedic	86/461 (19%)
Unknown	15/461 (3%)
Duration of work (years)	17.9 ± 11.89
Physician	13.24 ± 11.85
Nurse	21.35 ± 9.71
Paramedic	15.29 ± 10.19
Place of work	
Hospital	385/461 (83.5%)
Primary health care center	76/461 (16.5%)

**Table 2:** Descriptive analysis for each question of the questionnaire. Abbreviations: Y/N: Yes/No; CDC: Centers for Disease Control and Prevention; COVID-19: coronavirus disease 2019; SARS: severe acute respiratory syndrome; WHO: World Health Organization.

Question	*n* (%)
Q 3.1 Have you ever heard about COVID-19 (Y/N)	457/4 (99/1)
Q.3.2 What is your source of information for SARS-CoV-2?	
Physician	130 (28.1%)
TV/Radio	322 (69.8%)
Internet/web pages/blogs	290 (63%)
Web page of Greek CDC	95 (20.6%)
Other	49 (10.6%)
Q 4. Can SARS-CoV-2 be transmitted sexually?	
Fully agree	31/461 (6.8%)
Agree	61/461 (13.2%)
Disagree	153/461 (33.2%)
Fully disagree	116/461 (25.3%)
Uncertain	99/461 (21.5%)
Q.5 Can SARS-CoV-2 be transmitted by the consumption of foods?	
Fully agree	67/461 (14.5%)
Agree	176/461 (38.1%)
Disagree	108/461 (23.4%)
Fully disagree	39/461 (8.4%)
Uncertain	67/461 (14.5%)
Q 6. Can SARS-CoV-2 be transmitted by respiratory droplets?	
Fully agree	277/461 (60%)
Agree	168/461 36.4%)
Disagree	1/461 (0.002%)
Fully disagree	1/461 (0.002%)
Uncertain	11/461 (2.4%)
Q 7. Is COVID-19 a cause for serious illness and death?	
Fully agree	152/461 (32.9%)
Agree	242/461 (52.5%)
Disagree	50/461 (10.8%)
Fully disagree	5/461 (0.010%)
Uncertain	9/461 (0.019%)
Q 8. Can symptoms of COVID-19 be similar with those of seasonal flu?	
Fully agree	119/461 (25.8%)
Agree	290/461 (63%)
Disagree	16/461 (3.5%)
Fully disagree	1/461 0.002%)
Uncertain	33/461 (7.1%)
Q 9. Do you know the recommendations of WHO for COVID-19?	
Yes	349/461 (76%)
No	59/461 (12.7%)
Uncertain	49/461(10.6%)
Q 10. Are the recommendations by the Greek health authorities on COVID-19 sufficient?	
Fully agree	145/461 (31.4%)
Agree	115/461 (24.9%)
Disagree	130/461 (28.2%)
Fully disagree	35/461 (7.5%)
Uncertain	30/461 (6.5%)
Q 11. Is there an available specific drug therapy for COVID-19?	
Yes	25/461 (5.5%)
No	336/461 (73%)
Uncertain	97/461 (21%)
Q 12. Is there an available vaccine for COVID-19?	
Yes	13/461 (2.8%)
No	385/461 (83.5%)
Uncertain	63/461 (13.6%)
Q 13. Do you believe that washing of hands reduces the risk of infection from SARS-CoV-2?	
Fully agree	189/461 (41%)
Agree	245/461 (53.2%)
Disagree	14/461 (3%)
Fully disagree	3/461 (0.7%)
Uncertain	10/461 (2.1%)
Q 14. If you received special advice by the hospital infectious committee for COVID-19 would you follow them?	
Yes	452/461 (98%)
No	7/461 (1.5%)
Q 15. How often do you wash your hands when at work? (Frequency)	
Often	79/461 (17.1%)
Very often	265/461 (57.4%)
Before and after contact with the patient/patient’s environment	115/461 (24.9%)
Few times	1/461 (0.002%)
At the end of work	1/461 (0.002%)
Q 16. Will you be vaccinated for SARS-CoV-2?	
Yes	200/461 (43.3%)
No	145/461 (31.4%)
Uncertain	110/461 (23.9%)
Q 17. Do you support the temporary traveling restrictions ban for the prevention of SARS-CoV-2?	
Fully agree	118/461 (25.5%)
Agree	210/461 (45.5%)
Disagree	86/461 (18.6%)
Fully disagree	10/461 (2.2%)
Uncertain	37/461 (8.2%)
Q 18. How do you judge your level of knowledge about COVID-19?	
Sufficient	242/461 (52.5%)
Inadequate	196/461 (42.5%)
Other	23/461 (5%)

**Table 3:** Knowledge towards preventive practices for SARS-CoV-2 by variables.

Variable		Knowledge *n* (%) or Mean ± SD																	
		Can SARS-Cov-2 Be Transmitted Sexually?		Can SARS-Cov-2 Be Transmitted by the Consumption of Foods?		Can SARS-Cov-2 Be Transmitted by Respiratory Droplets?		Is the COVID-19 A Cause for Serious Illness and Death?		Can Symptoms of COVID-19 Be Similar with Those of Seasonal Flu?		Is There an Available Specific Drug Therapy for COVID-19?		Is There an Available Vaccine for SARS-Cov-2?		Do You Believe That the Washing of Hands Reduces the Risk of Infection from SARS-Cov-2?		Knowledge Score	
		Fully Agree/Agree	Disagree/Fully Disagree/Uncertain	Fully Agree/Agree	Disagree/Fully Disagree/Uncertain	Fully Agree/Agree	Disagree/Fully Disagree/Uncertain	Fully Agree/Agree	Disagree/Fully Disagree/Uncertain	Fully Agree/Agree	Disagree/Fully Disagree/Uncertain	Yes	No/Uncertain	Yes	No/Uncertain	Fully Agree/Agree	Disagree/Fully Disagree/Uncertain	Low Score *n* = 54	High Score *n* = 407
**sex**	**Male**	70/118 (59.3%)	48/118 (40.7%)	43/119 (36.1%)	76/11 (63.9%)	113/118 (95.7%)	5/118 (4.3%)	96/118 (81.3%)	22/118 (18.7%)	107/119 (89.9%)	12/119 (10.1%)	19/118 (16.2%)	99/118 (83.8%)	9/119 (7.5%)	110/119 (92.5%)	114/119 (95.7%)	5/119 (4.3%)	11/119 (9.3%)	108/119 (90.7%)
	**Female**	198/336 (58.9%)	141/336 (41.9%)	94/337 (27.5%)	243/337 (72.5%)	331/339 (97.6%)	8/339 (2.4%)	297/339 (87.6%)	42/339 (12.4%)	302/339 (89%)	37/339 (11%)	102/339 (30%)	237/339 ^!^ (70%)	67/341 (19.6%)	274/341 ^”^ (80.4%)	319/340 (93.8%)	21/340 (6.2)	43/341 (12.7%)	298/341 (87.3%)
**Age (years)**		43.70 ± 10.72	44.64 ± 10.91	44.93 ± 11.06	43.77 ± 10.67	44.10 ± 10.67	45.53 ± 14.40	44.51 ± 10.50	42.26 ± 12.36	44.22 ± 10.81	43.93 ± 10.80	45.25 ± 10.33	43.75 ± 10.94	43.44 ± 10.55	44.35 ± 10.84	44.31 ± 10.80	43.04 ± 10.28	44.22 ± 11.93	44.20 ± 10.64
**Work duration (years)**		17,66 ± 11.08	18 ± 11.23	17.87 ± 11.41	17.77 ± 11.07	17.80 ± 11.12	20.29 ± 13.94	18.11 ± 11.08	16.42 ± 11.62	17.87 ± 11.26	17.50 ± 10.63	18.67 ± 11.21	17.50 ± 11.15	16.80 ± 11.12	18.06 ± 11.20	17.92 ± 11.21	17.31 ± 10.81	17.89 ± 11.70	17.85 ± 11.14
**Occupation**	**Nurse**	124/213 (58.2%)	89/213 (41.8%)	68/216 (31.5%)	148/216 (68.5%)	213/217 (98.1%)	4/217 (1.9%)	198/217 (91.2%)	19/217 (8.8%)	195/216 (90.3%)	21/216 (9.7%)	69/215 (32%)	146/215 (68%)	47/218 (21.5%)	171/218 (78.5%)	207/218 (95%)	11/218 (5%)	28/218 (12.8%)	190/218 (87.3%)
	**Physician**	95/140 (67.9%)	45/140 (32.1%)	55/139 (39.6%)	84/139 (60.4%)	139/141 (98.5%)	2/141 (1.5%)	110/139 (79.2%)	29/139 (20.8%)	132/141 (93.7%)	9/141 (6.3%)	13/141 (9.2%)	128/141 (90.8%)	7/141 (5%)	134/141 (95%)	136/140 (97.1%)	4/140 (2.9%)	5/141 (3.6%)	136/141 (96.4%)
	**Paramedics**	40/86 (46.6%)	46/86 * (53.4%)	20/86 (23.3%)	^ 60/86 (69.7%)	81/84 (96.4%)	3/84 (3.6%)	72/86 (83.7%)	14/86 ^#^ (16.3%)	69/86 (80.3%)	17/86 ^@^ (19.7%)	31/86 (36%)	55/86 ^~^ (64%)	18/86 (21%)	68/86 (79%)	76/86 (88.4%)	10/86 ^&^ (11.6%)	14/86 (16.3%)	72/86 ^+^ (83.7%)

* *p* = 0.013, ^ *p* = 0.034, ^$^ *p* = 0.005, ^#^ *p* = 0.012, ^@^ *p* = 0.014, ^!^ *p* = 0.010, ^~^ *p* < 0.001, ” *p* = 0.008, ^&^ *p* = 0.045, ^+^ *p* = 0.009.

**Table 4:** Attitudes towards preventive practices for SARS-CoV-2 by variables.

Variable		Attitudes *n* (%) or Mean ± SD						
		Q16. Will You Be Vaccinated against Sars-Cov-2?				Attitudes Score		
		Yes	No/Uncertain	Fully Agree/Agree	Disagree/Fully Disagree/Uncertain	0	1	2
**sex**	**Male**	69/118 (58.5%)	49/118 (41.5%)	74/119 (62.2%)	45/119 (37.8%)	19/119 (15.9%)	57/119 (47.9%)	43/119 (36.1%)
	**Female**	131/336 (38.98%)	205/336 * (61%)	254/341 (74.5%)	87/341 ^@^ (25.5%)	56/341 (16.4%)	184/341 (54%)	101/341 (29.6%)
**Age (years)**		43.18 ± 11.4	44.76 ± 10.18	44.76 ± 11.54	43.258 ± 11.03	45.20 ± 10.30	43.17 ± 10.74	44.73 ± 11.10
**Work duration (years)**		16.27 ± 11.82	18.87 ± 10.48 ^^^	18.29 ± 11.58	16.81 ± 11.18	19.72 ± 11.05	17.59 ± 10.74	17.40 ± 11.98
**Specialty**	**nurse**	73/215 (34%)	142/215 (66%)	166/218 (76.2%)	52/218 (23.8%)	36/218 (16.5%)	142/218 (65.1%)	40/218 (18.4%)
	**physician**	85/140 (60.7%)	55/140 (39.3%)	82/141 (58.2%)	59/141 (41.8%)	26/141 (18.4%)	63/141 (44.7%)	52/141 (36.9%)
	**Paramedic**	37/85 (43.5%)	48/85 ^#^ (56.5%)	66/86 (76.7%)	20/86 * (23.3%)	13/86 (15.1%)	43/86 (50%)	30/86 (34.9%)
**Occupational facility**	**Hospital**	164/380 (43.2%)	216/380 (56.8%)	280/385 (72.7%)	105/385 (27.3%)	64/385 (16.6%)	198/385 (51.4%)	123/385 (31.9%)
	**Primary health care center**	30/61 (49.2%)	31/61 (50.8%)	37/62 (59.6%)	25/62 ^!^ (40.4%)	10/62 (16.1%)	37/62 (59.6%)	15/62 (24.3%)

* *p* = 0.001 between groups, ^ *p* = 0.019 between groups, ^#^ *p* < 0.001 between groups, ^@^ *p* = 0. 011, ^!^ *p* = 0.036.

**Table 5:** Practices towards performing preventive practices for SARS-CoV-2 by variables.

Variable		Practices *n* (%) or Mean ± SD						
		Q14. Will You Follow Special Advices for Covid-19?		Q15. How Often Do You Wash Your Hands at Work?		Practice Score		
		Yes	No/Uncertain	After Every Patient	Often/Very often/Few Times/At the End of Work	0	1	2
**Sex**	**Male**	116/119 (97.5%)	3/119 (2.5%)	19/119 ^^^ (16%)	100/119 (84%)	3/119 ^~^ (2.5%)	97/119 ^~^ (81.5%)	19/119 ^~^ (16%)
	**Female**	336/340 (98.8%)	4/340 (1.2%)	95/339 (28.1%)	244/339 (71.9%)	4/341 ^~^ (1.1%)	243/341 ^~^ (71.2%)	94/341 ^~^ (27.5%)
**Age (years)**		44.36 ± 10.74	38.20 ± 8.40	43.80 ± 10.02	44.30 ± 11.06	33.85 ± 10.10 ^$^	44.56 ± 10.95 ^$^	43.77 ± 10.06 ^$^
**Work duration (years)**		17.99 ± 11.21 ^#^	11.0 ± 4.12 ^#^	16.73 ± 10.36	18.18 ± 11.45	9.33 ± 5.50	18.37 ± 11.45	16.80 ± 10.39
**Specialty**	**Nurse**	215/217 (99%)	2/217 (1%)	56 (25.80%) *	161 (74.20%) *	2/218 ^@^ (1%)	161/218 ^@^ (73.8%)	55/218 ^@^ (25.2%)
	**Physician**	139/141 (98.6%)	2/141 (1.4%)	48 (34.2%) *	92 (65.72%) *	2/141 ^@^ (1.4%)	91/141 ^@^ (64.5%)	48/141 ^@^ (34%)
	**Paramedics**	84/86 (97.6%)	2/86 (2.4%)	10 (8.8%) *	79 (92%) *	2/86 ^@^ (2.3%)	76/86 ^@^ (90.7%)	8/86 ^@^ (9.3%)
**Occupational facility**	**Hospital**	379/384 (98.7%)	5/384 (1.3%)	96/383 (25%)	287/383 (75%)	5/385 (1.3%)	285/385 (74%)	95/385 (24.7%)
	**Primary health care center**	61/62 (98.4%)	1/62 (1.6%)	18/62 (29.1%)	44/62 (70.9%)	1/62 (1.7%)	43/62 (69.3%)	18/62 (29%)

* *p* < 0.001 between groups (nurses vs. physicians vs. paramedics), ^^^ *p* = 0.027, ^#^ *p* = 0.016, ^~^ *p* < 0.001 between groups, ^@^ *p* = 0.005 between groups, ^$^ *p* = 0.030 between groups.

**Table 6:** Association between knowledge, attitude, and practices scores.

Variable		Attitudes Score			Practices Score		
		0	1	2	0	1	2
**Knowledge Score**	**Poor level**	15/54 (27.77%)	30/54 (55.55%)	9/54 (16.66%)	4/54 (7.40%)	44/54 (81.49%)	6/54 (11.11%)
	**High level**	61/407 (14.98%)	211/407 (51.85%)	135/407 (33.17%) *	3/407 (0.74%)	297/407 (72.97%)	107/407 (26.29%) ^^^
**Practice Score**	**0**	2/7 (28.57%)	5/7 (71.43%)	0/7 (0%)			
	**1**	63/341 (18.47%)	163/341 (47.80%)	115/341 (33.73%)			
	**2**	11/113 (9.74%)	73/113 (64.60%)	29/113 (25.66%) ^$^			

* *p* = 0.011, ^^^ *p* < 0.001, ^$^ *p* = 0.009.

## Supplementary Materials

The following are available online at https://www.mdpi.com/1660-4601/17/14/4925/s1, Table S1: questionnaire.
