# Supplementary material for: Assessment of Knowledge, Attitudes, and Practices towards New Coronavirus (SARS-CoV-2) of Health Care Professionals in Greece before the Outbreak Period
Source: Int J Environ Res Public Health. 2020 Jul 8;17(14):4925. doi: 10.3390/ijerph17144925 (PMC7400230; doi:10.3390/ijerph17144925)
Supplement: Supplementary file 1 [file ijerph-17-04925-s001.pdf]

University of Thessaly
School of Health Sciences
Department of Nursing
Department of Respiratory Medicine, Medical School,
University Hospital of Larissa.

K.A.P STUDY FOR THE RECENT EPIDEMIC OF (2019-nCoV) IN HEALTH CARE PROFESSIONALS

DEMOGRAPHICS

**Age (years): …….**	**Sex: male** ☐<br>**female** ☐	**Nationality: …………………**	**Municipal: ……………………….**<br>**Work place:**<br>**Hospital** ☐<br>**Primary Health care center** ☐

**Occupation:**	**Physician** ☐	**Nurse** ☐	**Paramedics** ☐
**Years at work…………..**			

**1. (Q1) Have you travelled at the past 6 months out of Greece?**	☐ **Yes**<br>☐ **No**	**If Yes country/countries ……………………………..**<br>**………………………………………………………………….**

**2. (Q2) Have been travelled familiar person at the past 6 months out of Greece?**	☐ **Yes**<br>☐ **No**	**If Yes country/countries ……………………………..**<br>**…………………………………………………………………………….**

**3. (Q.3.1) H What is your source of information for SARS-CoV-2?**	☐ **Yes**	**Q.3.2 If yes source of information;**<br>☐ **Physician** ☐ **Web/Web pages/blogs**<br>☐ **TV/Radio** ☐ **Web page of Hellenic CDC**<br>**Other-please specify …………………………………………..**
	☐ **No**	

4. (Q4) Can SARS-CoV-2 be transmitted sexually?

☐ Fully agree ☐ Agree ☐ Disagree ☐ Fully disagree ☐ Uncertain

5. (Q5) Can SARS-CoV-2 be transmitted by the consumption of foods?

☐ Fully agree ☐ Agree ☐ Disagree ☐ Fully disagree ☐ Uncertain

6. (Q6) Can SARS-CoV-2 be transmitted by respiratory droplets?

☐ Fully agree ☐ Agree ☐ Disagree ☐ Fully disagree ☐ Uncertain

7. (Q7) Is the COVID-19 a cause for serious illness and death?

☐ Fully agree ☐ Agree ☐ Disagree ☐ Fully disagree ☐ Uncertain

8. (Q8) Can these symptoms of COVID-19 be similar with those of seasonal flu?

☐ Fully agree ☐ Agree ☐ Disagree ☐ Fully disagree ☐ Uncertain

9. (Q9) Do you now the recommendations of WHO for COVID-19?

Yes ☐ No ☐ ☐ Uncertain

10. (Q10) Are the recommendations by the Greek health authorities on covid-19 sufficient?

☐ Fully agree ☐ Agree ☐ Disagree ☐ Fully disagree ☐ Uncertain

11. (Q11) Is there an available specific drug therapy for COVID-19? ☐ Yes ☐ No ☐ Uncertain

12. (Q12) Is there an available vaccine for COVID-19? ☐ Yes ☐ No ☐ Uncertain

13. (Q13) Do you believe that the washing of hands reduces the risk of infection from SARS-CoV-2?

☐ Fully agree ☐ Agree ☐ Disagree ☐ Fully disagree ☐ Uncertain

14. (Q14) If you received special advices by the Hospital infectious committee for COVID-19 would you follow them?	☐ Yes
	☐ No

15. (Q15) How often do you wash your hands when at work?

☐ Often ☐ Very often ☐ Before and after contact with the patient/patient's environment ☐ Few times ☐ At the end of wok

16. (Q16) Will you be vaccinated for SARS-CoV-2?

☐ Yes ☐ No ☐ I am not answer

17. (Q17) Do you support the ban of travelling for countries with increase cases of SARS-CoV-2?

☐ Fully agree ☐ Agree ☐ Disagree ☐ Fully disagree ☐ Uncertain

18. (Q18) How do you judge your level of knowledge about COVID-19

☐ Sufficient ☐ Inadequate ☐ Other: ....

Thank you for your cooperation.
